# An ammonite trapped in Burmese amber

**DOI:** 10.1073/pnas.1821292116

**Published:** 2019-05-13

**Authors:** Tingting Yu, Ulysses Thomson, Lin Mu, Andrew Ross, Jim Kennedy, Pierre Broly, Fangyuan Xia, Haichun Zhang, Bo Wang, David Dilcher

**Affiliations:** ^a^State Key Laboratory of Palaeobiology and Stratigraphy, Nanjing Institute of Geology and Palaeontology, Chinese Academy of Sciences, Nanjing 210008, China;; ^b^CAS Center for Excellence in Life and Paleoenvironment, Chinese Academy of Sciences, Nanjing 210008, China;; ^c^School of Earth and Space Sciences, University of Science and Technology of China, Hefei 230026, China;; ^d^School of Earth Sciences, University of Bristol, Bristol BS8 1TQ, United Kingdom;; ^e^Department of Natural Sciences, National Museum of Scotland, Edinburgh EH1 1JF, United Kingdom;; ^f^Oxford University Museum of Natural History, Oxford OX1 3PW, United Kingdom;; ^g^Department of Earth Sciences, University of Oxford, Oxford OX1 3AN, United Kingdom;; ^h^Private address, 59840 Lompret, France;; ^i^Lingpoge Amber Museum, Shanghai 201108, China;; ^j^Key Laboratory of Zoological Systematics and Evolution, Institute of Zoology, Chinese Academy of Sciences, Beijing 100101, China;; ^k^Department of Geology and Atmospheric Science, Indiana University, Bloomington, IN 47405

**Keywords:** amber, ammonite, fossil, paleoecology, taphonomy

## Abstract

Amber is fossilized tree resin, and inclusions usually comprise terrestrial and, rarely, aquatic organisms. Marine fossils are extremely rare in Cretaceous and Cenozoic ambers. Here, we report a record of an ammonite with marine gastropods, intertidal isopods, and diverse terrestrial arthropods as syninclusions in mid-Cretaceous Burmese amber. We used X-ray–microcomputed tomography (CT) to obtain high-resolution 3D images of the ammonite, including its sutures, which are diagnostically important for ammonites. The ammonite is a juvenile *Puzosia* (*Bhimaites*) and provides supporting evidence for a Late Albian–Early Cenomanian age of the amber. There is a diverse assemblage (at least 40 individuals) of arthropods in this amber sample from both terrestrial and marine habitats, including Isopoda, Acari (mites), Araneae (spiders), Diplopoda (millipedes), and representatives of the insect orders Blattodea (cockroaches), Coleoptera (beetles), Diptera (true flies), and Hymenoptera (wasps). The incomplete preservation and lack of soft body of the ammonite and marine gastropods suggest that they were dead and underwent abrasion on the seashore before entombment. It is most likely that the resin fell to the beach from coastal trees, picking up terrestrial arthropods and beach shells and, exceptionally, surviving the high-energy beach environment to be preserved as amber. Our findings not only represent a record of an ammonite in amber but also provide insights into the taphonomy of amber and the paleoecology of Cretaceous amber forests.

## Significance

Aquatic organisms are rarely found in amber, but when they occur they provide invaluable evidence for the better understanding of amber taphonomy and past ecosystems. We report an ammonite and several marine gastropods alongside a mixed assemblage of intertidal and terrestrial forest floor organisms in mid-Cretaceous Burmese amber. Our discovery indicates that the Burmese amber forest was living near a dynamic and shifting coastal environment. The ammonite also provides supporting evidence for the age of the amber, which is still debated, and represents a rare example of dating using fossils present inside the amber.

Amber provides a unique mode of preservation for organisms, and when inclusions are present they are usually 3D fossils of terrestrial plants, microorganisms, arthropods, and even vertebrate remains (1–3). Amber deposits are therefore considered to be exceptional Lagerstätten, providing unique windows into past ecosystems (4–6). Given that amber is formed by the fossilization of terrestrial plant resins, the capture of marine inclusions may be considered extremely rare. However, some recent findings of marine and freshwater fossils, particularly, microfossils such as diatoms, radiolarians, ostracods, and copepods, have provided fresh insights into amber taphonomy (7–12).

Burmese amber (from northern Myanmar) contains the most diverse biota of all known Cretaceous ambers (13, 14). Over the last 100 years, and particularly in the past two decades, Burmese amber has received worldwide scientific interest; more than 500 families of invertebrates, vertebrates, protists, plants, and fungi have been reported (15). Here, we provide an account of an exceptional piece of amber that preserves a unique assemblage of marine macrofossils, alongside intertidal, fully terrestrial, and possibly freshwater aquatic arthropods.

## Results

**Fig. 1.**
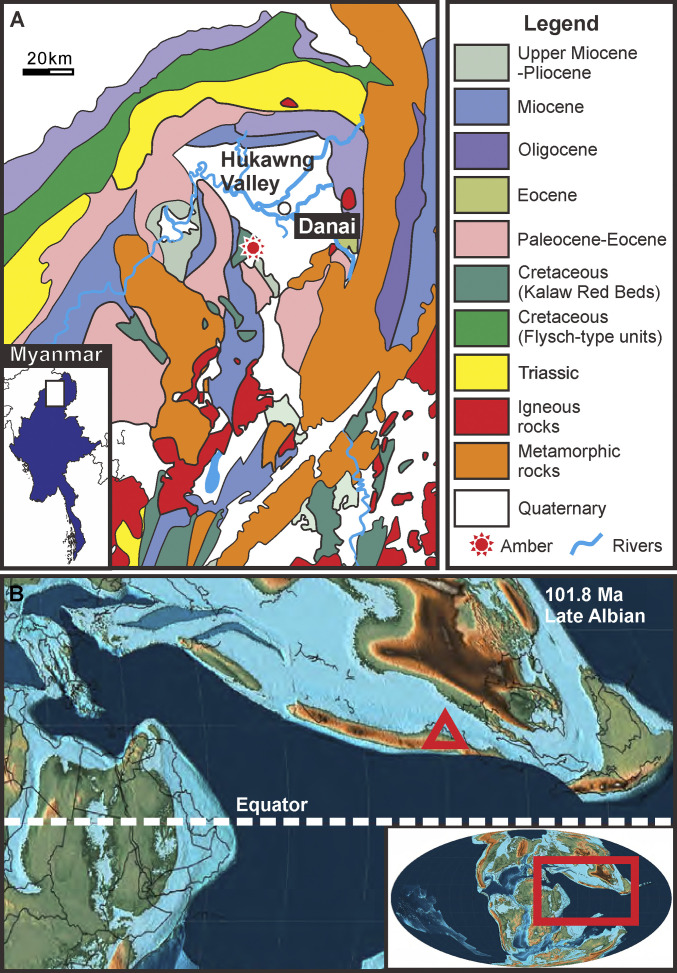
Geological and paleogeographic maps of Burmese amber. (*A*) Geological map showing the position of Burmese amber in Hukawng Valley, northern Myanmar. (*B*) Paleogeographic map showing the position (red triangle) of Burmese amber site during Late Albian (14, 17).

The ammonite-bearing piece of amber (BA18100) was obtained from an amber mine located near Noije Bum Village, Tanaing Town (ref. 16 and Fig. 1). It is 33 mm long, 9.5 mm wide, and 29 mm high, and its weight is 6.08 g. There is a diverse assemblage (at least 40 individuals) of arthropods in this amber sample that live today in both terrestrial and marine habitats. Of the terrestrial fauna, Acari (mites) are the most abundant, with 23 specimens; also present are Araneae (spiders), Diplopoda (millipedes), and representatives of the insect orders Blattodea (cockroaches), Coleoptera (beetles), Diptera (true flies), and Hymenoptera (wasps). The arthropod assemblage consists mostly of forest floor-dwelling taxa, and living representatives are generally associated with leaf litter or the top layers of soil. There are several isopods preserved which are consistent with littoral or supralittoral taxa. In addition to the ammonite itself, four definitively marine gastropod shells and one putatively marine isopod are present.

### Ammonite.

**Fig. 2.**
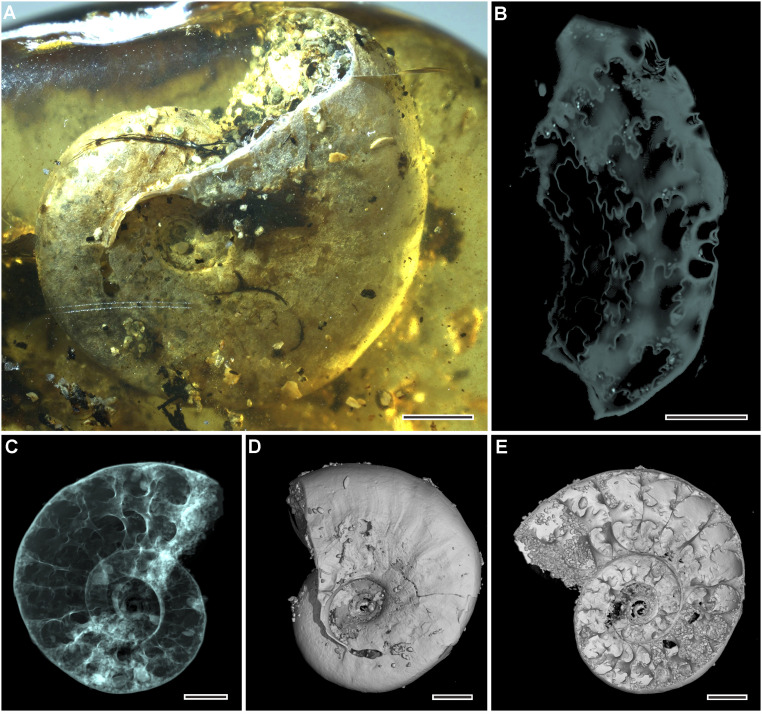
Ammonite *Puzosia* (*Bhimaites*) Matsumoto. (*A*) Lateral view under light microscopy. (*B*) Flattened sutures reconstructed by microtomography. (*C*) Microtomographic reconstruction, apparent view. (*D*) Microtomographic reconstruction, surface rendering; (*E*) Microtomographic reconstruction, virtual section. (Scale bars, 2 mm.)

The ammonite is a juvenile (the adapertural septa are not crowded), has a maximum preserved diameter of 12 mm, and appears to retain the original aragonitic shell, on the basis of its appearance in reflected light (Fig. 2*A*). It is composed in part by the body chamber, but the apertural part is damaged, as revealed by the survival of a 60° sector of the umbilical wall extending beyond the fragment of the inner flanks of the shell (Fig. 2). Coiling is moderately involute, with ∼64% of the previous whorl covered. The small, shallow umbilicus comprises 18% of the diameter, the low umbilical wall is very weakly convex, and the umbilical shoulder is broadly rounded. The whorl section is compressed, with a whorl breadth-to-height ratio of around 0.7 (the specimen has undergone some postmortem deformation). The inner flanks are very weakly convex, the outer flanks flattened and weakly convergent, the ventrolateral shoulders broadly rounded, and the venter very weakly convex. Ornamentation consists of low falcoid folds, lirae, and riblets that are prorsiradiate and very weakly concave on the inner flank, flexing back and weakly convex at midflank before flexing forward and weakly concave on the outer flank, sweeping forward over the ventrolateral shoulders and crossing the venter in a broad convexity. The suture of the penultimate whorl, revealed in X-ray–microcomputed tomography, is only partially decipherable. E/A is broad, bifid, and moderately incised; A is narrower and possibly trifid; and A/U2 is narrow, little incised, and bifid (Fig. 2*B*; see Movie S1 for detailed account).

Given the age of the amber (discussed below), a compressed, involute, weakly ornamented ammonite could belong to one of three principal groups, the superfamilies Phylloceratoidea, Lytoceratoidea, or Desmoceratoidea. The visible structure of the sutures and the lack of distinctive even lirae eliminate the first two superfamilies from consideration. Within Desmoceratoidea, compressed weakly ornamented taxa comparable to the present specimen occur in two subfamilies of the family Desmoceratidae, the Beudanticeratinae and Puzosiinae.

**Fig. 3.**
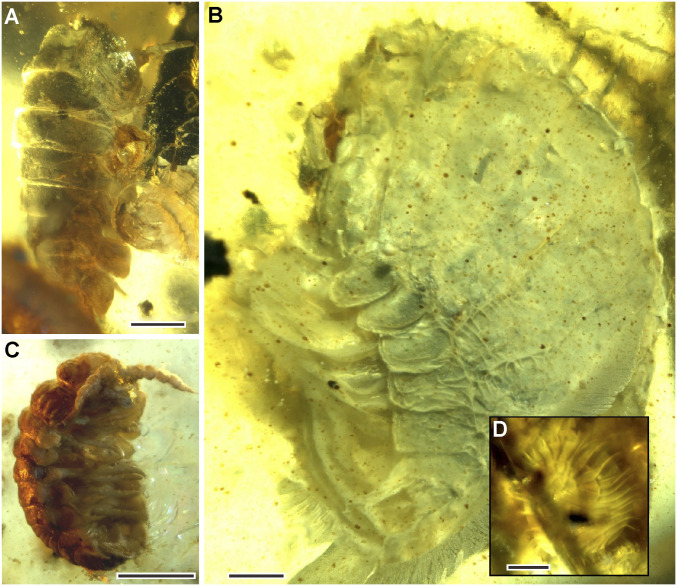
Isopods of uncertain taxonomic affinity, but generally consistent with littoral or supralittoral taxa. (*A*) Isopod 1. (*B*) Isopod 2. (*C*) Isopod 3. (*D*) Circular structure attached to the dorsal surface of isopod 2. (Scale bars, 1 mm in *A* and *C*. Scale bar, 0.5 mm in *B* and *D*.)

Among the Beudanticeratinae, a possible assignation is to *Beudanticeras* Hitzel (18), a genus that ranges from the Lower to Upper Albian and is known from Europe, North Africa, the Middle East, KwaZulu-Natal (South Africa), Mozambique, Madagascar, northern Pakistan, Australia, Patagonia, and Antarctica. There are similarities with juveniles of the Lower Albian *Beudanticeras caseyi* Collignon (ref. 19, p. 72, pl. 267, fig. 1165; holotype refigured by ref. 20, text-fig. 3j, k), known from Madagascar and northern KwaZulu-Natal (South Africa), and comparably sized juveniles of the Tunisian upper Lower Albian *Beudanticeras dupinianum var. africana* (ref. 21, p. 133, pl. 5, figs. 16, 17; text-fig. 49), as figured by Latil (ref. 22, pl. 3, figs. 1–19). These species, however, do not develop the low folds and undulations of the present specimen, and *Beudanticeras* is restricted to the Albian. Among the Puzosiinae, there are similarities with *Puzosia* (*Bhimaites*) Matsumoto (23), which ranges from the Upper Albian to the Upper Cenomanian and is known from western Europe, North Africa, Angola, KwaZulu-Natal (South Africa), Madagascar, South India, Japan, and Venezuela. The falcoid course of the ornament, which matches our specimen, is seen in several representatives of the genus, for example, the Upper Albian *Puzosia* (*Bhimaites*) *pinguis* (24) illustrated by Kennedy and Klinger (ref. 25, text-fig. 12a–g). A feature of *Puzosia* (*Bhimaites*) is the development of constrictions on the internal mold; their position is marked on the shell surface by much weaker depressions and associated collar ribs. These are very weakly expressed or absent in specimens comparable in size to the present specimen [see, for example, the somewhat larger (30 mm diameter) individual of *Bhimaites stoliczkai* (26) figured by Renz (ref. 27, pl. 8, fig. 2)]; this species ranges from the Upper Albian to Lower Cenomanian.

To conclude, features of the ammonite preserved most strongly suggest a juvenile *Puzosia* (*Bhimaites*), a subgenus that first appeared in the Upper Albian and ranged through the Cenomanian.

### Isopods.

There are four isopod specimens in the amber (Fig. 3 *A*–*C*) and a further three specimens, which cannot be determined but may also be isopods. The first isopod (Fig. 3*A*) is consistent with terrestrial isopods in body shape: the eyes appear to be reduced, although this is not entirely clear, and there are six to seven pereonite segments with all pereopods ambulatory. The form of the uropods, if present, is not entirely clear, which is unfortunate, as this is a key character for distinguishing marine and terrestrial taxa. It is similar to Armadillidae, which is recorded from Burmese amber (28), but also exhibits characters of marine taxa, such as having a larger posterior part, but many important characters are obscured, so it is difficult to identify with certainty. Although Armadillidae is generally considered to be terrestrial, Poinar (28) considered that the features present in his fossil excluded it from those Oniscoidea mostly adapted to terrestrial habitats, such as the strictly terrestrial *Myanmariscus*, also recorded from Burmese amber (29).

The second isopod (Fig. 3*B*) exhibits an elongated exopod uropod and is similar to extant Sphaeromatidae in general habitus, indicating that it is possibly a marine or intertidal isopod. Sphaeromatids are typically marine, but many have been known to occur in estuaries (30) and intertidal zones and even to extend into freshwater habitats, including karstic streams and caves (31). The pale coloration and reduced eyes could indicate a stygobiont, but the color could also be taphonomic. These characters are also not restricted to cave dwellers, as some open-marine isopods also exhibit them (32). There is a peculiar circular structure seemingly attached to this specimen (Fig. 3*D*), although the association could be taphonomic.

The other two isopods (e.g., Fig. 3*C*) exhibit characters of terrestrial or supralittoral isopods and are possibly associated with the extant Oniscidea: Tylidae. The uropods appear to be reduced, as is typical of the more terrestrial taxa, and the visible antennae are thick at the base with strong basal segments tapering gradually toward the apex with a two- or three-jointed flagellum. The first pair of antennae appear to be strongly reduced, which is also an indication of the Oniscidea, with Tylidae only retaining the proximal article (33).

There are a few other specimens that are probably isopods, including one that is badly damaged with most of its ventral side obscured by a gastropod. The coloration and coxal plates are similar to those of the specimen shown in Fig. 3*A*, but there is a partial eye preserved, and it is larger. It is closely associated with a gastropod, but this is probably taphonomic. There are two other very badly damaged specimens, which may be isopods and two others, which may be isopod larvae, but they are poorly preserved.

Although taxonomic assignment is difficult based on specimens in which key characters cannot be observed, the specimens present in the piece of amber seem to be consistent with littoral or supralittoral isopods, with one possible fully marine species.

### Gastropods.

**Fig. 4.**
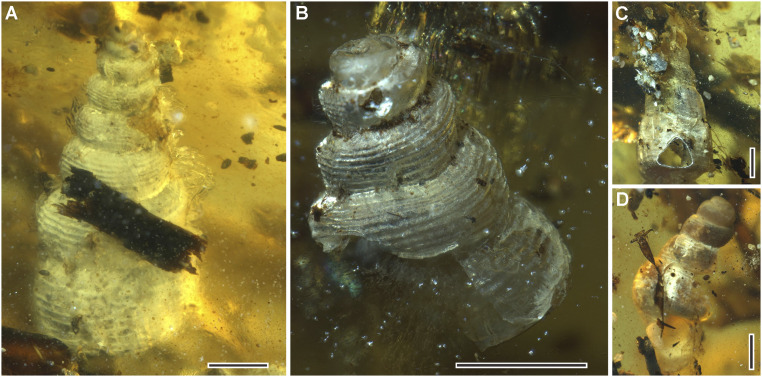
Gastropods. (*A*) *Mathilda* sp. (*B*) *Mathilda* sp. (*C*) Undetermined specimen. (*D*) Undetermined specimen. (Scale bars, 1 mm.)

Four marine gastropod shells are also preserved with the ammonite (Fig. 4), of which two are well-preserved and can be attributed to the genus *Mathilda* Semper (Mathildidae) by the small, conical shell with heterostrophic protoconchs, whorl sides rounded and basally subcarinate, base broadly arched, and ornament of strong spiral cords and fine axial threads (34). *Mathilda* was mainly distributed in the western Tethys sea during the Cretaceous, and our fossils are a Cretaceous record of this genus from the eastern Tethys sea.

### Terrestrial Arthropod Assemblage.

**Fig. 5.**
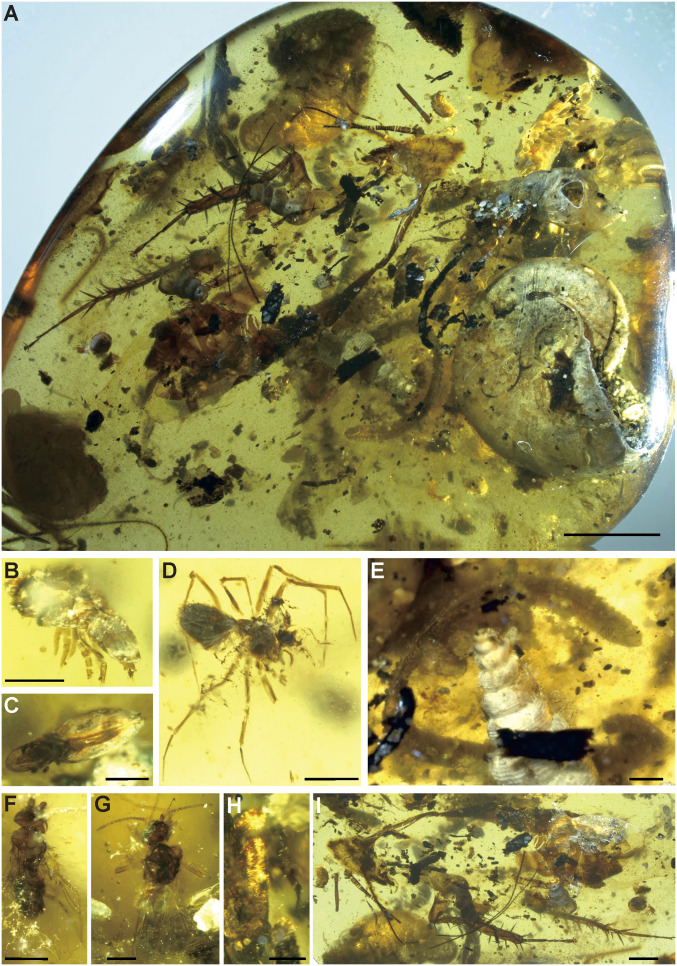
Amber inclusions. (*A*) Amber piece showing most large inclusions. (*B*) Acari: Phthiracaridae. (*C*) Acari: Euphthiracoidea. (*D*) Araneae: Oonopidae. (*E*) Diplopoda. (*F*) Diptera: Phoridae. (*G*) Hymenoptera: Chrysidoidea. (*H*) Coleoptera. (*I*) Blattodea. (Scale bar, 5 mm in *A*. Scale bars, 1 mm in *E* and *H*. Scale bars, 0.5 mm in *B*–*D*, *F*, and *G*. Scale bar, 2 mm in *I*.)

There are 22 oribatid mites in the piece, most of which are ptyctimous, meaning they can close their prodorsum over their legs as protection against predators (box mites). Most of the mites (15 individuals) are similar in appearance to Phthiracaridae (Fig. 5*B*), but the ventral shields would need to be examined to clarify this, which is difficult given their position in the amber (Fig. 5*A*). They have previously only been described from Baltic amber and younger deposits. Also, although obscured in the piece, there appear to be representatives of Euphthiracaroidea (Fig. 5*C*), based on the fusion of the plates, and some Brachypylina.

Ptyctimous oribatids are common in soil/forest floor communities and are usually considered to be secondary decomposers/fungivores, although some ptyctimous mites are known to be xylophagous and feed on dead wood as primary decomposers (35). Either way, they are generally found in the presence of decaying plant material (36, 37). Oribatids are generally free living in the upper soil layers, but Phthiracaroidea are commonly found within fallen leaves or conifer needles (37), and ground-dwelling species can be found up to 4 m from the ground on live tree trunks (38) with a clear gradient of community species composition ascending the trunk (39).

There is one spider preserved (Fig. 5*D*) that is unfortunately partly decomposed, and the eyes and chelicerae are not well preserved, so it is difficult to identify, but it is similar in general appearance to some Cretaceous Oonopidae which have been found in amber from Canada and Myanmar (40, 41). Oonopidae (goblin spiders) are described by Penney (40) as wandering, active predators, fast moving and nocturnal, and are known from a varied range of habitats (42), including the forest floor or tree bark (43).

There are 12 adult insects preserved in the piece, eight of which are true flies (Diptera), two are beetles (Coleoptera), one is a parasitic wasp (Hymenoptera), and one is a cockroach (Blattodea). There are also several larval specimens. Diptera is mostly represented by small nematoceran midges or gnats (Ceratopogonidae, Cecidomyiidae, or Chironomidae), and there are two small brachyceran hump-backed flies with cyclorrhaphan-type antennae and wing venation (Fig. 5*F*) consistent with scuttleflies (Phoridae). Some nematoceran midges (e.g., chironomids) have aquatic larvae, which are usually found in freshwater habitats, but others (ceratopogonids and cecidomyiids) could have terrestrial or plant gall stages. One of them may be a gall midge that could have been associated with trees, similar to the parasitoid wasp (Fig. 5*G*), which belongs to Chrysidoidea.

There are two beetles preserved in the amber, but they are largely obscured by other material. The larger one (Fig. 5*H*) is obscured by a gastropod, but the characters that can be seen include hind and mid legs with the femur expanded; tibiae narrow at the base, expanding toward apex; thick tibial spines reaching at least the length of the first tarsomere; five-segmented tarsi, gradually reducing in length from the first to the fifth, with each expanding in width from base to apex; ring of clumped hairs around the apice of the tibiae and each tarsomere, except the fifth that has two claws; and fore leg curved in a raptorial style. The pronotum is transverse; the elytra are distorted but appear oval-shaped and may have a black-and-white–banded color pattern; the head (and antennae) are either not preserved or are obscured by a gastropod shell.

The cockroach (Fig. 5*I*) is about 20 mm long, with most of the head, thorax, and abdomen missing, but the general overall shape is preserved. Also, many important characters, such as the antennae, maxillary and labial palps, and the right fore, mid, and hind legs are preserved. There are no remains of the wings. The maxillary palps are long; the head appears quite narrow, but not entirely preserved. The partial preservation suggests that not all of the cockroach became engulfed in the resin, and the exposed parts decomposed before it was covered by another layer. Many cockroaches are found in forest floor, leaf litter habitats, and many are found associated with decaying wood, although others are arboreal or aquatic (44).

One millipede is preserved (Fig. 5*E*) that is around 15 mm long, very slender, and has relatively short legs. The amber around the millipede is cloudy, so it is difficult to observe characters in detail, certainly important characters such as the organ of Tömösváry or ozopores. Millipedes are important detritivores and are mainly forest floor dwellers and are considered to have been for their whole evolutionary history (45). The body form of the specimen probably matches the “borer” (platydesmoid) type suggested by Kime and Golovatch (46), which suggests that it inhabited the leaf litter or uppermost soil layers (stratobiont) or that it was an underbark xylobiont (47).

## Discussion

U-Pb dating of zircons from the volcanoclastic matrix of the amber has given a maximum age of 98.8 ± 0.6 Ma (48), which places it in the Early Cenomanian based on the 100.5 ± 0.4 Ma age assigned to the base of the stage by Cohen et al. (49); however, this dates the amber-bearing horizon, not the amber itself. This age is incompatible with the record of the exclusively Upper Albian ammonite *Mortoniceras*, which was found in a sandstone above primary Burmese amber deposits (16). The specimen of this ammonite was neither described nor figured, and we could not examine it, as attempts to locate the specimen have not been successful; therefore we cannot confirm its identity. Thus, the incompatibility of the age and the *Mortoniceras* ammonite remains unresolved. The presence of borings of martesine bivalves in the outer rim of pieces of Burmese amber suggested that the amber could be older than the age of the bed it was collected from. Bivalves have also been found within the amber and therefore bored into it while the amber was still soft and are thus similar in age to the bed (50).

Amber pieces can be reworked and redeposited in younger deposits; therefore, dating amber is sometimes controversial. The amber-bearing strata can be dated from palynofloras, ammonites, and radiodating evidence, but the amber could be older. Marine inclusions can help date ambers, as marine diatoms and other marine microfossils supported an Albian–Cenomanian age of Charentese ambers of France (7, 51). The present discovery is another interesting example of dating using fossils present inside the amber.

How did the amber that would have flowed from a tree capture both terrestrial (insects, millipedes, spiders, and mites) and marine (ammonite, gastropods, and isopods) organisms? Analysis of the depositional environment supports the model of an estuarine, coastal landscape for the mid-Cretaceous amber forests. Poinar et al. (52) analyzed Burmese amber and found that the most likely origin of the resin which formed the amber was araucarian conifers (but see ref. 53), which can be closely associated with coastal habitats. Many pieces of Burmese amber were bored by martesine pholadid bivalves, indicating that the amber was deposited in a brackish nearshore environment (50). Martesine bivalves have also been found within the amber, indicating that the resin was still soft when the bivalves started boring into it, which suggests that resin-producing trees were growing near to the site of deposition. The ammonite and gastropods had suffered damage before entombment. For example, the ammonite had lost at least a 60° sector of its body chamber, indicating that this was not the shell of a live individual. There is no evidence of any soft-part preservation of any of the gastropods, which also suggests that these were dead shells. The amber also contains some shell sand.

Of the many thousands of specimens of Burmese amber studied, only one ammonite is known. It is an exceptional occurrence and may record an exceptional event. There are three possible scenarios: (*i*) There was a sandy beach with resin-producing trees growing very close. The terrestrial insects were trapped in the resin while it was still on the tree, and as it traveled down the tree trunk it picked up the lower-lying terrestrial arthropods, such as the mites. When it reached the ground (the beach?) it landed on the sand and shells, trapping the supralittoral isopods as they traversed the beach. As these forests are considered to have been coastal, this scenario could have been commonplace, but the probability of such amber pieces surviving would be slight, owing to the dynamic nature of beaches, which would explain the rarity of such pieces of amber in the fossil record. (*ii*) There was a tsunami that flooded the amber-producing forest, bringing marine debris into the forest and thus into contact with numerous blobs of resin. This would certainly be an exceptional event, although it could possibly be expected that more diverse marine inclusions, including ones with soft-bodied preservation, would be found in the amber, if this scenario were true. (*iii*) Being a tropical environment, it could be assumed that tropical storms were fairly common and could therefore blow seashells and sand inland. This could also account for the martesine bivalve shells being found within the amber. However, if this was a fairly common event, it could be expected that occurrences of marine shells in amber would be more common as well.

Marine and terrestrial organisms may get trapped in a single resin piece located at the edge of a coastal forest, and more complicated scenarios such as liquid resin with sea water contact are not needed (6, 11, 54, 55), especially as Schmidt and Dilcher (8) found that resin barely solidifies when submerged in water. The incomplete preservation and lack of the soft body of the marine ammonite (Fig. 2*A*) and four gastropods (Fig. 4) indicate that they were dead and had experienced abrasion by the sea on the seashore before they were engulfed by resin. Moreover, the aperture of the ammonite is filled with coarse shell sand, which is also present in other parts of the amber piece (Fig. 5*A*), suggesting that the resin-producing trees were very close to the coast. Therefore, we consider that the first scenario is the most likely. Other marine inclusions in the future may suggest other scenarios, although it is possible for all of the scenarios above to have happened over the lifetime of the amber-producing forest. It seems clear, however, that the forest was living near a dynamic and ever changing coastal environment.

## Conclusions

It is rare to find aquatic organisms in amber, and it is extremely rare to find marine organisms in amber, let alone macroscopic marine organisms mixed with intertidal, terrestrial, and potentially freshwater aquatic organisms. The exceptional occurrence of macroscopic marine macrofossils in the resin suggests that the amber forest was growing close to a coast, possibly next to a beach, and could have been subjected to exceptional events. The shells may record an exceptionally high, perhaps storm-generated tide, or even a tsunami or other high-energy event. Alternatively, and more likely, the resin fell to the beach from coastal trees, picking up terrestrial arthropods and beach shells and, exceptionally, surviving the high-energy beach environment to be preserved as amber.

## Materials and Methods

The amber piece (BA18100) is deposited in the Lingpoge Amber Museum in Shanghai. Photographs were taken using a Zeiss AXIO Zoom.V16 microscope system at the State Key Laboratory of Paleobiology and Stratigraphy, Nanjing Institute of Geology and Paleontology, Chinese Academy of Sciences (NIGPAS). In most cases, incident and transmitted light were used simultaneously. All images are digitally stacked photomicrographic composites of ∼40 individual focal planes that were obtained using the software Helicon Focus 6 (http://www.heliconsoft.com) for better illustration of the 3D structures, as described by Schmidt et al. (56).

To three-dimensionally reconstruct the ammonite, we scanned the fossil at the micro-CT laboratory of NIGPAS, using a 3D X-ray microscope (3D-XRM), Zeiss Xradia 520 versa. Unlike conventional micro-CT, which relies on maximum geometric magnification and a flat panel detector to achieve high resolution, 3D-XRM uses CCD-based objectives to achieve higher spatial resolution. Based on the size of the fossil specimen, a CCD-based 0.4× objective was used, providing isotropic voxel sizes of 13.36 μm with the help of geometric magnification. During the scan, the acceleration voltage for the X-ray source was 70 kV (current 86 μA), and a thin filter (LE3) was used to avoid beam-hardening artifacts. To improve signal-to-noise ratio, 2001 projections over 360° were collected, and the exposure time for each projection was 3 s. Volume data processing was performed using software VGStudio Max (version 3.0; Volume Graphics). The Nonplanar Clipping function program (VGStudio version 3.0) was used to reconstruct the suture along the curved surface of the ammonite.

## Supplementary Material

Supplementary File

Supplementary File
