# Supplementary material for: An ammonite trapped in Burmese amber
Source: Proc Natl Acad Sci U S A. 2019 May 13;116(23):11345–50. doi: 10.1073/pnas.1821292116 (PMC6561253; doi:10.1073/pnas.1821292116)
Supplement: Supplementary File [file pnas.1821292116.sapp.pdf]

Video S1 Legend: Flattened sutures reconstructed by microtomography.
